# An Iranian Patient with Maroteaux Type Acromesomelic Dysplasia, Showing no Involvement of Distal Lower Limbs

**DOI:** 10.4274/jcrpe.galenos.2019.2019.0090

**Published:** 2020-03-19

**Authors:** Hossein Moravej, Mozhagn Moghtaderi, Sara Mostafavi

**Affiliations:** 1 Neonatal research center, Shiraz University of Medical Sciences, Shiraz, Iran; 2 Department of Pediatric Endocrinology, Shiraz University of Medical Sciences, Shiraz, Iran; 3 Student Research Committee, Shiraz University of Medical Sciences, Shiraz, Iran

## Dear Editor,

Acromesomelic dysplasia, Maroteaux type (AMDM) is an autosomal recessive form of acromesomelic dysplasia characterized by disproportionately short stature, shortening of the middle and distal segments of the limbs as well as vertebral involvement. AMDM is the result of a mutation in the natriuretic peptide receptor 2 (NPR2) genes which impairs skeletal growth (1,2,3).

**Figure 1:**
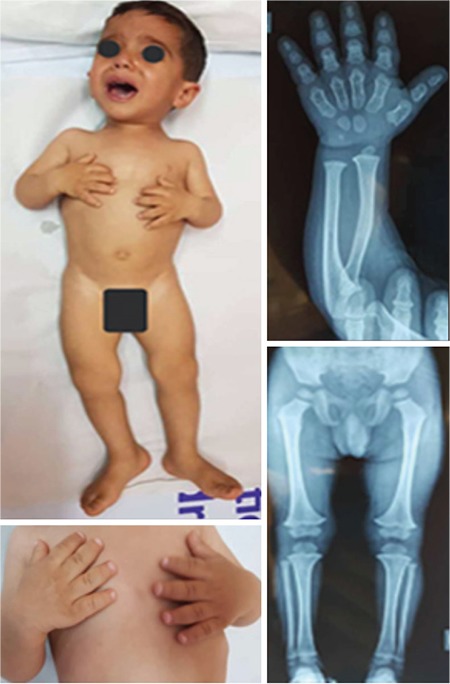
Clinical characteristics and radiographic features of the patient. Frontal bossing, low set ears and wrist joint hyperflexibility as well as short and broad fingers of the hand with small nails are noteworthy. Radiographic findings showed radial bowing with posterior dislocation, short lower end of the ulna as compared to the radius, and broad metacarps with wide phalanges

A 2- years old boy, offspring of non-consanguineous parents and of a 2^nd^ pregnancy, was referred to the endocrine and metabolic center of the Nemazee Hospital, located in southwestern Iran, for evaluation of short stature. The patient was born at 38 weeks of gestation by cesarean section and was healthy by Apgar scoring. Birth weight was 3100 g, length 45 cm and head circumference 35 cm. He had no dysmorphic features and general physical examination revealed no pathology. There was no satisfactory length gain after birth, as noticed by his parents. At the referral time at age 2 years the patient had a weight of 8200 g (-4 SD), a length of 71 cm [-4 standard deviation (SD)]. Head circumference was 48 cm (0.3 SD). Fingers of the hand were extremely short and broad with small nails; there was no redundant skin on the fingers (Figure 1). His feet and toes were normal. Frontal bossing, low set ears and wrist joint hyperflexibility were prominent features. All developmental milestones were within normal limits. His older sibling was of normal stature. Matental height was 156 cm (-1.6 SD) and the father was 163 cm (-1.9 SD) tall. His older sibling was of normal stature. None of the other family members were affected.

Radiographic findings showed curved radius, relatively short ulna, and broad metacarps with wide phalanges. The vertebrae were of normal size and showed no beaking. Iliac wings and metatarsal bones were normal (Figure 1). DNA was extracted from the peripheral blood by standard techniques and microsatellite analyses were performed. Whole exon sequencing test and mutation confirmation by direct Sanger screening were performed and evaluated by reference sequence, AMDM maps to 9p13.3. Cytogenic evaluation could not be performed in the parents and in the older sibling. Informed consent was obtained from his parents for this report.

The mutation of the case was displayed in NPR2 with cytogenic location of 9p13.3. This mutation overlaps with two diseases: firstly, autosomal dominant epiphyseal chondrodysplasia, miura type which is characterized by tall stature, long hands and feet with arachnodactyly, and secondly, short-rib thoracic dysplasia 5 with or without polydactyly (4,5). Both diseases have completely different clinical patterns and radiographic manifestations from AMDM.

In summary, considering the skeletal changes, radiological findings and sequence analysis of the mutation, this patient is the first AMDM case reported from Iran. The patient had severe short stature, but no obvious abnormality in the distal segment of his lower limb. We suggest that this patient may represent a new variant form of AMDM.
